# 5-HT modulation of pain perception in humans

**DOI:** 10.1007/s00213-017-4686-6

**Published:** 2017-08-10

**Authors:** Sarah L. Martin, Andrea Power, Yvonne Boyle, Ian M. Anderson, Monty A. Silverdale, Anthony K. P. Jones

**Affiliations:** 1 The Human Pain Research Group, Faculty of Biology, Medicine and Health, University of Manchester, Manchester, UK; 2 Division of Neuroscience and Experimental Psychology, School of Biological Science, Faculty of Biology, Medicine and Health, University of Manchester, Manchester Academic Health Science Centre, Manchester, UK; 3 GlaxoSmithKline Clinical Unit Cambridge, Addenbrooke’s Centre for Clinical Investigation, Addenbrooke’s Hospital, Cambridge, UK; 4 Neuroscience and Psychiatry Unit, University of Manchester, Manchester Academic Health Science Centre, Manchester, UK; 5 Department of Neurology, Salford Royal NHS Foundation Trust, Manchester Academic Health Science Centre, M6 8HD, Salford, UK

**Keywords:** Serotonin, Tryptophan, Pain, Acute tryptophan depletion (ATD)

## Abstract

**Introduction:**

Although there is clear evidence for the serotonergic regulation of descending control of pain in animals, little direct evidence exists in humans. The majority of our knowledge comes from the use of serotonin (5-HT)-modulating antidepressants as analgesics in the clinical management of chronic pain.

**Objectives:**

Here, we have used an acute tryptophan depletion (ATD) to manipulate 5-HT function and examine its effects of ATD on heat pain threshold and tolerance, attentional manipulation of nociceptive processing and mood in human volunteers.

**Methods:**

Fifteen healthy participants received both ATD and balanced amino acid (BAL) drinks on two separate sessions in a double-blind cross-over design. Pain threshold and tolerance were determined 4 h post-drink via a heat thermode. Additional attention, distraction and temperature discrimination paradigms were completed using a laser-induced heat pain stimulus. Mood was assessed prior and throughout each session.

**Results:**

Our investigation reported that the ATD lowered plasma TRP levels by 65.05 ± 7.29% and significantly reduced pain threshold and tolerance in response to the heat thermode. There was a direct correlation between the reduction in total plasma TRP levels and reduction in thermode temperature. In contrast, ATD showed no effect on laser-induced pain nor significant impact of the distraction-induced analgesia on pain perception but did reduce performance of the painful temperature discrimination task. Importantly, all findings were independent of any effects of ATD on mood.

**Conclusion:**

As far as we are aware, it is the first demonstration of 5-HT effects on pain perception which are not confounded by mood changes.

## Introduction

Pain is essential for avoiding damage and a vital learning mechanism for survival. The processing of pain is not isolated to one region of the brain and integrates information from a number of regions which form the pain matrix (Vogt et al. 1996; Legrain et al. 2011; Jones and Brown 2017; Denk et al. 2014). Hence, pain is considered to be the combination of multiple factors such as stimulus intensity, emotional and contextual state and individual past experiences which contribute to the expectation of pain. In contrast to acute pain, the development of chronic pain, particularly neuropathic pain, is regarded to be, in most cases, a maladaptive process providing the sufferer with no benefits, whereas in chronic musculoskeletal pain, there may be a mixture of pains, some of which have a protective function and others which are more maladaptive (Jones and Brown 2017). There is a high prevalence of chronic pain in the population and a limited choice of successful treatments. A potential choice of treatment is the use of selective serotonin reuptake inhibitors (SSRIs) which increase serotonin (5-HT) within the brain which are also a common choice of antidepressant. There have been mixed results reported from the use of SSRIs, and the mechanism of action on pain processing is unclear (Jung et al. 1997; Jann and Slade 2007). Evidence for the specific role of 5-HT in the modulation of pain processing arises mainly from animal studies. Descending inhibitory serotonergic spinal-raphe projections from the nucleus raphe magnus form an endogenous descending pain-modulating network, capable of concurrently mediating both the facilitation and inhibition of nociceptive processing (Basbaum and Fields 1984; Fields et al. 1991; Kwiat and Basbaum 1992; Millan 2002; Wei et al. 2012). In addition, ascending serotonergic pain pathways, projecting from the midbrain dorsal-raphe nucleus to limbic and adjacent forebrain sites, control the attentional processing of nociception (Boyle et al. 2008; Brown and Jones 2008; Kosofsky and Molliver 1987; O’Hearn and Molliver 1984).

The development of chronic pain has been linked with the so-called maladaptive neuroplastic changes in the brain which result in pain sensitivity (Jensen et al. 2016; Petersen-Felix and Curatolo 2002; Jones and Brown 2017; Apkarian et al. 2004; Baliki et al. 2008). A study by Nitsche et al. (2009) reported a role of 5-HT in neuroplasticity and reported that an acute increase of 5-HT in healthy subjects increased facilitatory plasticity. Therefore, 5-HT levels within the brain may also have an effect on the abnormal neuroplastic changes associated with chronic pain.

In humans, the exact role of 5-HT as a modulator in the perception of experimental or chronic pain remains unclear. Some evidence for the role of 5-HT in the pathophysiology of several human chronic pain disorders is based on the analgesic properties of 5-HT-modulating antidepressants (Dharmshaktu et al. 2012; Mika et al. 2013; Atkinson et al. 1999; Bomholt et al. 2005; Lee and Chen 2010; McQuay and Moore 1997). However, the precise mechanisms behind these analgesic effects have not been defined. It remains uncertain whether the apparent analgesic effects of 5-HT-modulating drugs are a direct consequence of increased availability of 5-HT or as a secondary effect of mood changes. Current arguments favour the direct effects of 5-HT, as analgesia generally occurs prior to any changes in mood (Hegerl et al. 2012; McQuay et al. 1996). A technique to experimentally investigate the action of 5-HT in humans is via global depletion of its precursor tryptophan (TRP) using a method known as acute TRP depletion (ATD).

Previously, research has shown how 5-HT levels can be manipulated to investigate pain processing via the supplementation and depletion of its precursor TRP (Wang et al. 2009; Carpenter et al. 1998; Chase et al. 2011). Previous findings show that TRP supplementation improves both pain tolerance and mood in healthy volunteers (Seltzer et al. 1982). In contrast, a study using ATD reported that there were no changes to the cold pressor pain (Abbott et al. 1992). However, the study discovered that morphine-induced analgesia was abolished by ATD and, hence, highlighting the complex involvement of serotonin in pain processing. Furthermore, the central processing of the pain caused from irritable bowel syndrome (IBS) has been further investigated using ATD research. A study showed that ATD in healthy subjects resulted in network disruption homologous to disruption seen in IBS sufferers with pain (Labus et al. 2011). Therefore, we hypothesised that depletion of TRP levels would result in the sensitisation of pain, which would be the opposite of supplementation of TRP studies.

Whilst there is still debate on the involvement of mood changes induced by the ATD (Benkelfat et al. 1994; Delgado et al. 1990; Ellenbogen et al. 1999; Robinson and Sahakian 2009), studies have successfully dissociated pain and mood changes following serotonin and noradrenaline supplementation in patients suffering from depression (Hegerl et al. 2012). A large-scale meta-analysis of 5-HT depletion studies has shown that mood is not affected within a healthy volunteer cohort with no history of depression (Young 2013). However, there is a slight negative change in mood within participants with a family history of major depressive disorder (MDD). Additionally, MDD sufferers in remission were reported to have the greatest decrease in mood, and serotonin depletion resulted in a relapse of MDD (Ruhé et al. 2007). As evidence suggests that ATD does not alter mood in subjects with no history of depression, for this study, we have excluded subjects with a history of depression and monitored alterations in mood throughout the study.

The role of 5-HT in pain perception is far from clearly defined; however, the use of SSRIs in treating pain conditions indicates that a low level of 5-HT may correlate with an increased sensitivity to pain (Goesling et al. 2013; Cooper et al. 2017). Therefore, the aim of this research was to use ATD to investigate the role of 5-HT in pain perception whilst attempting to dissociate the effects from mood changes. The study primarily investigated the participants’ threshold and tolerance level of a heat thermode after the ATD and control balanced (BAL) drink. An additional exploratory investigation used CO_2_ laser stimuli combined with an attention and distraction paradigm to explore the role of 5-HT in the central processing of pain perception. This study will provide further insight into how we utilise drugs to modulate 5-HT systems as pain therapies, independent of mood manipulation, as well as provide an insight into the attentional processing of pain in chronic pain conditions, such as fibromyalgia (FM) and complex regional pain syndrome (CRPS), which are associated with abnormal physiological levels of 5-HT (Alnigenis and Barland 2001).

## Methods

### Participants

Twenty healthy volunteers gave informed consent to participate in the study. Prior to study entry, a psychological assessment determined volunteer suitability. The exclusion criteria for recruitment included a history of depression, neurological or psychiatric illness (assessed using the research version of the SCID: Structured Clinical Interview for Diagnostic and Statistical Manual of Mental Disorders, fourth edition; DSM-IV; First et al. 2002), or having a first-degree relative with a history of depression. Further exclusion criteria consisted of recreational drug or excessive caffeine use, smokers and females with unpredictable menstrual cycles. Additional criteria for female volunteers controlled for mood, hormonal changes and pain sensitivity during their menstrual cycle (Menkes et al. 1994). A local research ethics committee approved the study (04/Q1407/286) and all participants gave written informed consent.

Although 20 volunteers participated in the study, two were excluded due to high HAD scores and three females were excluded due to the phase of their cycle (recruited prior to additional exclusion factors being enforced) Therefore, statistical analysis reports the data of 15 volunteers (6 males).

### Acute tryptophan depletion

TRP availability is the principal rate-limiting step in the 5-HT biosynthetic pathway (Ruddick et al. 2006). In humans, lowering central serotonergic activity by ATD is accomplished by consuming a TRP-free amino acid drink, which results in a substantial decline in plasma TRP levels (~70%), with peak depletion occurring approximately 4–6 h later (Moore et al. 2000). For this study, we used an ATD mixture and a balanced control mixture (BAL) which contained all amino acids. The compositions of the drinks were in replication of the methods used in Moore et al. (2000).

Prior to testing days, the participants were required to undertake a low-protein, caffeine-free, alcohol-free diet and fast from midnight. The participants were reminded via an email 2 days prior to each of their visits to ensure that they followed the pre-study instructions. In addition to this, the researcher verified that the participants complied with dietary instructions. Participants arrived at 9:00 am on testing day and were randomly assigned a 150-ml amino-acid drink. Due to lower body mass, females received 80% of the amino-acid cocktail. The time of this consumption was classed as time point 0 h, following which volunteers were exposed to a neutral environment (access to books and videos free from emotional content).

#### Procedure

Participants were asked to complete three visits (one screening session and two experimental sessions). The experimental sessions (visits 2 and 3) were each separated by at least 2 weeks for assessment of sensitivity to laser stimulation. This was increased to 4 weeks for female volunteers, allowing both experimental visits to coincide with the same phase of their menstrual cycle (i.e. if their first session occurred during the luteal phase of their cycle, they would return for the second session in the same phase), which controlled for pain and mood changes, associated with luteal phase dysphoric disorder (Menkes et al. 1994) and diminished serotonergic responses during the luteal phase of the menstrual cycle (Warner et al. 1991).

During experimental visits 2 and 3, paradigms A and B were assessed to investigate pain processing. Volunteers adhered to the aforementioned diet and consumed either an ATD drink or BAL drink consistent with the double-blind cross-over design of the experiment.

#### Visit 1—screening

The screening visit included assessments of participant suitability, training on experimental procedures and familiarisation with the rating scales to minimise variability between visits due to anxiety or task learning. Permission was obtained to test a urine sample for recreational and illegal drugs. No ATD was carried out during the screening visit, and participants organised visits 2 and 3 for the forthcoming weeks.

#### Visits 2 and 3—ATD pain investigation

##### Paradigm A

Pain threshold and tolerance were assessed at time points +4 and +6 h, measured using a Marstock heat thermode (THERMOTEST, Somedic Production AB, 19205 Sollentuna, Sweden) applied to the dorsum of the right forearm. To measure the pain threshold, the thermode temperature was adjusted from baseline (32 °C), using the multiple staircase method as previously described (Gracely et al. 1988), until a sensation of pain was perceived (‘pain threshold’). Subsequently, the temperature was further increased from the level established as ‘pain threshold’, until the pain became unbearable and ‘pain tolerance’ was recorded (maximum temp 52 °C).

##### Paradigm B

During paradigm B, pain was administered using a carbon dioxide (CO_2_) laser which is a technique consistent with similar published data from our group (Kulkarni et al. 2005; Boyle et al. 2008). The rapid nature of the laser stimulus means that it is ideal for observing attentional manipulation. The CO_2_ laser delivered radiant heat stimuli (beam 15 mm diameter, pulse 150 ms duration) to the dorsum of the right forearm. To prevent nociceptor sensitisation, the location of successive stimuli was moved around a 3 × 5-cm stimulation site on the dorsal surface of the forearm. The position of the stimulation site was identical for each experimental session. Throughout the study, volunteers wore protective goggles and earplugs to mask acoustic interference from the laser apparatus.

Initial psychophysics assessment determined the energy level required to elicit a moderately painful stimulus. An 11-point numerical scale was used to report pain perception (0–10; 0 = no sensation, 4 = just painful, 6 = moderately painful and 10 = unbearable pain). The main task of paradigm B included two variations: attention and distraction which are outlined below. Both variations were delivered in a two-block design of 30 laser stimuli delivered at 10 s intervals, and volunteers were asked to discriminate between two painful stimuli intensities (low and high intensity). These levels were determined as 90% (low) and 110% (high) of the laser energy (W/cm^2^) required to produce a pain score of 6 on the aforementioned 0–10 intensity scale. At the end of each recording block, volunteers were asked to rate the average pain experience.

The attention variant of paradigm B asked the volunteers to attend to the pain stimuli. In contrast, during the distraction task, volunteers were required to divert their attention away from the laser pain by calculating a series of 2-digit subtraction problems of varying difficulty (e.g. 71 − 51=, 92 − 27=). The calculation was displayed on a computer screen for 5 s, the laser pulse was delivered after 1 s and the volunteer was required to solve the calculation in the remaining 4 s. The percentage of correct mathematical solutions and correct rating of the pain level delivered was calculated.

In order to match tasks visually, the mathematical problem was presented to the volunteer in both the attention and distraction tasks. During the distraction task, volunteers were required to perform the subtraction, whereas in the attention task, volunteers were required to fixate on the screen, whilst discriminating between the two levels of heat intensity, but not attempt the calculation.

### Plasma tryptophan collection and assay

During visits 2 and 3, blood samples were collected (at time points 0, +4 and +6 h) in lithium heparin tubes and immediately centrifuged for 10 min at 2400 rpm/4 °C. Plasma was isolated and stored at −20 °C before being assayed for total and free TRP. In addition, the ratio of large nucleotide amino acids (LNAAs) and TRP was calculated as this is a well-known measure of the available TRP in the brain for serotonin synthesis (Fernstrom 1977). Plasma TRP and LNAA concentrations were measured by high-performance liquid chromatography with fluorescence end-point detection. The plasma TRP and LNAA concentrations at time points +4 and +6 h were averaged within subject to create the ‘post’-drink level.

### Psychological assessment

Mood was assessed throughout the two experimental days at set time points. The Hospital Anxiety and Depression Scale (HADS) score (Zigmond and Snaith 1983) was completed at time point 0 h at each visit. A visual analogue scale for depression (VAS; rated on a 100-mm line where 0 mm = none and 100 mm = extreme; Wewers and Lowe 1990) was completed at 0, +4 and +6 h. A Profile of Mood State (POMS) (where increased score correlates to increased depressive mood disturbance; McNair et al. 1971) questionnaire was also completed at time points 0, +4 and +6 h. Prior to starting the study, we were unsure as to which questionnaire would be more sensitive to changes in mood state; therefore, we selected three questionnaires. All three were reported to avoid selection bias.

### Analysis

Statistical analyses were performed using IBM SPSS Statistics 22 (SPSS Inc., Chicago, IL) and graphs created using GraphPad Prism (version 7). Statistical analysis for threshold and tolerance for paradigm A was carried out using repeated measures analysis of variance (RM-ANOVA) with within-subject factors: condition (BAL vs. ATD) and pain type (threshold vs. tolerance). Statistical analysis for paradigm B was carried out using RM-ANOVA with the following within-subject factors: condition (BAL vs. ATD), task (attending vs. distraction) and pain level (low and high). Furthermore, Spearman’s rank correlation analysis was carried out to establish the relationship between the percentage reduction of TRP levels and experimental output. Non-parametric analysis was carried out due to negatively skewed data. Statistical analysis was carried out on the HADS and POMS scores using RM-ANOVA analysis with within-subject factors: condition (BAL vs. ATD) and time point (0, 4 and 6 h). The correct ratings of the stimuli (sensory discrimination) were analysed using a RM-ANOVA with within-subject factors: condition (BAL vs. ATD) and pain level (low vs. high), and correct number of calculations during paradigm B were analysed using a two-tailed paired samples *t* test. Significance was considered at *p* < 0.05 and data was expressed as mean ± SD.

## Results

### Plasma tryptophan

**Fig. 1:**
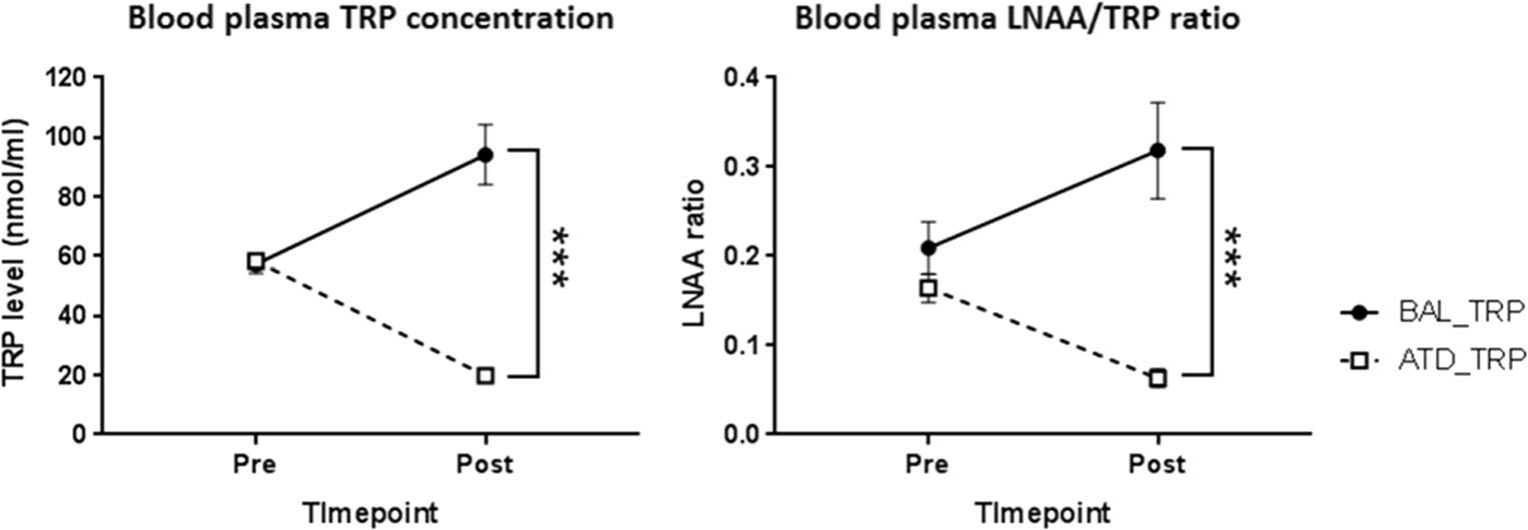
The change in TRP levels and the LNAA/TRP ratio pre- and post-depletion drink measured from blood samples at 0 h (pre) and average of 4 and 6 h (post). (*Left*) The average TRP level at 0 h was 57.35 ± 11.70 vs. 58.46 ± 6.49 nmol/ml for BAL and ATD conditions, respectively, which reported no significant difference (*p* = 0.7). The average TRP level post-drink was 94.20 ± 38.91 vs. 19.84 ± 4.36 nmol/ml for BAL and ATD conditions, respectively, with statistical difference (*p* < 0.001). (*Right*) The average LNAA:TRP ratio at 0 h was 0.21 ± 0.11 vs. 0.16 ± 0.06 for BAL and ATD, respectively. Post-drink, the LNAA:TRP ratio was reported as 0.32 ± 0,21 vs. 0.06 ± 0,04 for BAL and ATD, respectively, with a statistically significant difference (*p* = <0.001). *Black*: BAL drink, *white*: ATD drink. Mean ± SD, *n* = 15, ***p < 0.001

Plasma TRP levels were significantly lower following the consumption of the ATD in comparison to the BAL drink. Before administration of the drinks, TRP levels were not significantly different across both conditions (BAL vs. ATD at 0 h; 57.35 ± 11.70 vs. 58.46 ± 6.49, *p* = 0.7). Post-drink, ATD significantly reduced plasma TRP levels (BAL vs. ATD; 94.20 ± 38.91 vs. 19.84 ± 4.36 nmol/m; *p* < 0.001; Fig. 1).

In comparison to pre-drink levels of TRP, ATD effectively reduced total plasma TRP levels by 65.05 ± 7.29%, and the ratio of TRP to large neutral amino acids (TRP:LNAA) decreased by 53.8 ± 28.52% (from 0.16 ± 0.06 to 0.06 ± 0.04). In contrast, under control conditions (BAL), total TRP levels increased from pre-drink concentration by 67.28 ± 10.42%, and the ratio of TRP:LNAA increased by 85.0 ± 19.29% (from 0.21 ± 0.11 to 0.32 ± 0.21) (Fig. 1). The post-drink concentration of TRP (nmol/ml) was reported to be 94.20 ± 38.9 nmol/ml for BAL and 19.84 ± 4.4 nmol/ml for ATD (mean ± SD) indicating a 74.61 ± 13.4% difference post-drink. There was no effect of order of the condition (i.e. administration of ATD drink on visit 2 or 3) on the change in TRP levels or LNAA:TRP ratio.

### ATD and questionnaire measures

There were no effects of condition (BAL vs. ATD) on any of the mood scores. The participants’ initial mood prior to each experiment (0 h), recorded by HADS scores, did not differ between conditions or time point (BAL vs. ATD; 5.13 ± 3.37 vs. 4.93 ± 3.06; *p* = 0.68).). Additionally, the POMS scores and depression VAS scores (mm) recorded at 0, 4 and 6 h showed no statistical difference for condition, time point, nor interaction between conditions (BAL and ATD) and time points (0, 4, 6 h) **(**see Table 1).

**Table 1:** The scores for POMS and depression VAS (mm) are reported for both conditions (BAL and ATD) for three time points (0, 4 and 6 h). A repeated measures (RM) ANOVA reported no statistical significant interaction between condition and time point for either POMS or VAS scores

		Time			RM ANOVA condition * time point
		0 h	4 h	6 h	
POMS	BAL	4.27 ± 3.77	4.67 ± 3.04	3.40 ± 2.72	*F* = 0.523, *p* = 0.599
	ATD	4.33 ± 3.70	3.87 ± 3.80	4.13 ± 4.34	
VAS (/100 mm)	BAL	5.73 ± 8.59	7.93 ± 10.25	6.80 ± 8.44	*F* = 1.248, *p* = 0.303
	ATD	4.26 ± 5.56	4.86 ± 5.07	6.93 ± 8.14	

### Paradigm A: pain threshold and tolerance using contact thermode

**Fig. 2:**
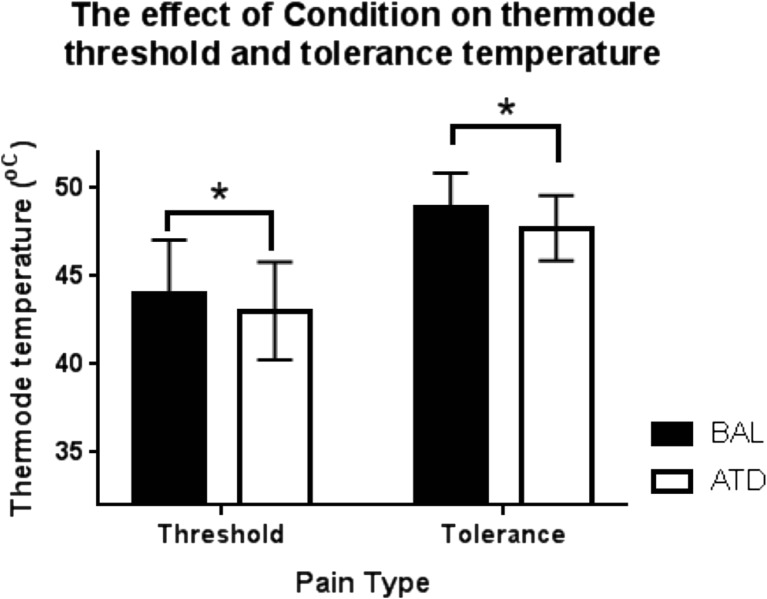
The mean thermode threshold and tolerance temperature plotted for all participants under BAL and ATD conditions. The threshold and tolerance of the thermode during the ATD condition were significantly reduced in comparison to the BAL condition (*F* = 7.111, *p* = 0.019). The *y*-axis is restricted to the minimum (32 °C) and maximum (52 °C) of the thermode temperature (*n* = 15, mean + SD)

A RM-ANOVA reported a significant effect of condition (BAL vs. ATD) (*F* = 7.111, *p* = 0.019), such that ATD led to a reduced threshold and tolerance to thermode temperature. The threshold and tolerance temperature was higher in the BAL condition (threshold 44.00 ± 3.06 °C, tolerance 48.87 ± 2.00 °C) in comparison to the ATD condition (threshold 43.03 ± 2.77 °C, tolerance 47.73 ± 1.86 °C) (Fig. 2). There was no effect of order (*F* = 0.428, *p* = 0.524). There was no significant condition * pain type interaction (*F* = 0.001, *p* = 0.979) indicating that the temperature for threshold and tolerance was affected in the same way by the ATD condition.

### Correlation analysis

**Fig. 3:**
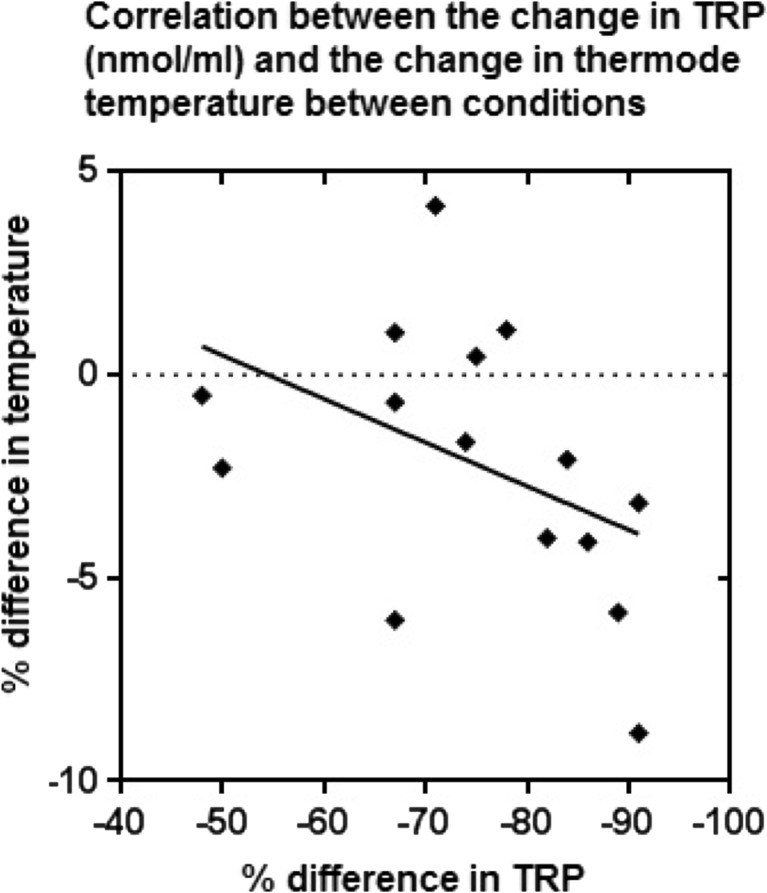
Correlation analysis comparing the post-drink difference in TRP (nmol/ml) for BAL and ATD and the average thermode temperature change for BAL to ATD. A significant positive correlation was reported via a Spearman’s rank correlation analysis, *r*_s_(13) = 0.548, *p* < 0.005, hence indicative that a higher decrease in TRP concentration correlates to a higher decrease in thermode temperature. A line of best fit is shown, *N* = 15

For the correlation analysis for each participant, a percentage difference was calculated of the post-drink TRP concentration (nmol/ml) after the ATD drink in comparison to post-BAL drink (−75.61 ± 13.40%) (mean ± SD). Given that ATD significantly reduced both threshold and tolerance temperatures, hence, an average thermode temperature was calculated per participant [(threshold + tolerance)/2] (group mean BAL 46.43 ± 43 °C, ATD 45.38 ± 2.23 °C) (mean ± SD) and the percentage difference in temperature between BAL and ATD post-drink calculated for each participant (group mean 2.21 ± 3.31%) (mean ± SD). Spearman’s rank correlation analysis showed a positive correlation between the difference of TRP level and the degree of change in the thermode temperature, *r*_s_(13) = 0.548, *p* < 0.005 (Fig. 3).

### Paradigm B: attention and distraction

#### CO_2_ laser pain scoring

The energy of the laser stimuli (W/cm^2^) administered at 90% (BAL 7.18 ± 1.81 to ATD 6.80 ± 1.87) or 110% (BAL 9.01 ± 2.19 to ATD 8.53 ± 2.16) of high pain was not significantly different between BAL and ATD conditions.

#### Attention and distraction paradigm

**Fig. 4:**
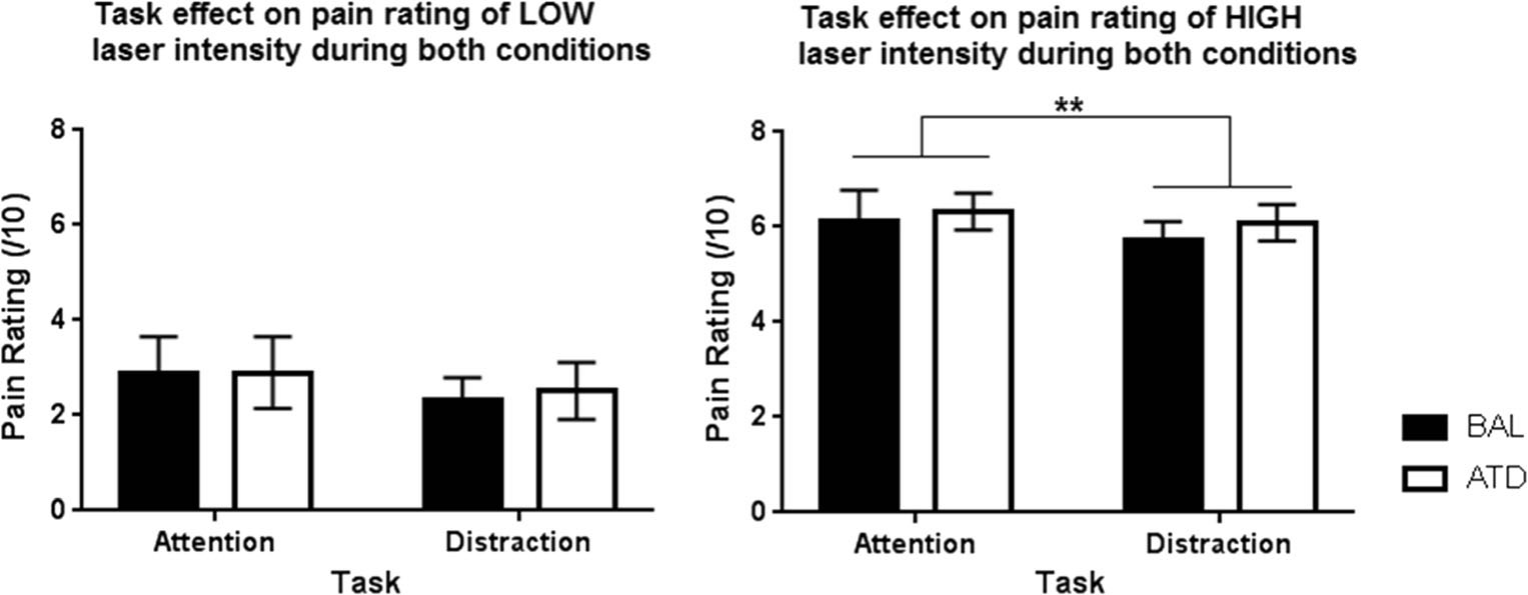
The effect of attention and distraction (task) on the pain rating for low (*left*) and high (*right*) laser-induced pain. (*Left*) A plot of the average pain rating participants gave for low-laser intensity during both task states. BAL or ATD condition did not report any significant difference in ratings of low pain (*F* = 0.33, *p* = 0.574). Distraction did not significantly reduce the rating of low pain in either condition; however, a trend was reported such that ATD caused an increase in pain ratings (*F* = 4.046, *p* = 0.065). There were no effects of condition on task pain ratings of low-laser intensity (*F* = 0.571, *p* = 0.463). (*Right*) A graph of the average pain rating participants gave for high-laser stimuli during the task states under both conditions. A significant reduction in pain rating of the high-laser stimuli was reported during distraction for both BAL and ATD conditions (*F* = 12.893, *p* = 0.003), yet no significant interaction of condition and task was reported (*F* = 0.875, *p* = 0.315). Hence, ATD did not affect the task-induced changes in pain ratings. *n* = 15, mean ± SD, **p < 0.01. Although not significant, a trend was shown for the pain rating under ATD conditions being slightly higher in the distraction task in comparison to the BAL condition (*F* = 3.663, *p* = 0.078)

Within-subject RM-ANOVA was conducted to compare the effect of condition (BAL and ATD) and task (attention and distraction) on the rating of the low- and high-laser stimuli. The overall pain ratings of low and high stimuli during both tasks were not significantly affected by the ATD condition (low: *F* = 0.33, *p* = 0.574; high: *F* = 3.663, *p* = 0.078). However, the high-laser stimuli did show a trend to significance (*p* = 0.078) such that the ATD resulted in an increase in pain ratings across both tasks, suggesting a slight increased sensitivity to the laser stimuli. Secondly, there was an effect of task such that pain ratings were lower during distraction compared to no distraction; for the low-laser intensity, this was a trend reduction (*F* = 4.046, *p* = 0.065), whereas for the high-laser stimuli, it was significant (*F* = 12.893, *p* = 0.003). There were no interactions between condition and task for low- (*F* = 0.571, *p* = 0.463) or high- (*F* = 0.875, *p* = 0.315) laser stimuli, indicating that the condition of the participant did not alter the effect of task-induced changes on pain rating (Fig. 4).

#### Cognitive performance

The ability to discriminate high- and low-intensity laser stimuli was calculated during paradigm 2 with a RM-ANOVA with within-subject factors: condition (BAL vs. ATD) and pain level (low vs. high). The condition * pain level interaction was significant (*F* = 7.28, *p* = 0.018) with participants significantly more likely to incorrectly rate low-intensity stimuli as high intensity under ATD conditions in comparison to BAL.

The percentage correct calculations during the distraction task of paradigm B were compared between BAL and ATD conditions by a two-tailed paired samples *t* test. The statistical analysis showed a significant reduction (*p* = 0.039) in correct calculations during distraction under the ATD condition.

## Discussion

The present study examined the role of 5-HT in human pain processing by depleting plasma TRP concentrations in healthy volunteers via ATD. Blood analysis indicated a significant reduction in total plasma TRP levels and the LNAA ratio which was consistent with previous findings (Gallagher et al. 2003; van der Veen et al. 2007). The LNAAs and TRP compete for uptake into the brain, and hence, the LNAA ratio was calculated to establish the degree to which TRP levels have decreased within the brain. Therefore, alongside the reduction in the TRP concentration in the blood plasma, the decline in the LNAA/TRP ratio reinforces that serotonin synthesis within the brain has been reduced via the ATD drink and repletion via the BAL drink. The observable rise in TRP levels under control conditions (BAL) is due to TRP repletion and is consistent with previous findings (Gallagher et al. 2003).

First of all, the analysis of the HADS and POMS questionnaires showed no significant difference between conditions (BAL vs. ATD), and hence, a dissociation between mood and the findings regarding pain perception can be made. This also supports the use of a strict exclusion criteria preventing the recruitment of participants whom are susceptible to 5-HT-induced mood changes (Benkelfat et al. 1994; Delgado et al. 1990; Moore et al. 2000; Hughes et al. 2003; Klaassen et al. 1999).

### Paradigm A: thermode threshold and tolerance

An interesting finding of this study was the significant reduction in the participants’ threshold and tolerance level of the heat thermode during ATD condition. To the best of our knowledge, this is the first time that this relationship has been reported, and indicates that TRP depletion results in an increased sensitivity to heat pain. Previous studies have highlighted how increasing the level of TRP within the brain causes a reduced sensitivity to pain (Seltzer et al. 1982), and hence, this study has reported the corresponding opposing effect as decreasing TRP levels has increased pain sensitivity.

Furthermore, the pain tolerance of an individual could be said to have a motivational component, and there has been research exploring the role of 5-HT in motivation and resilience. For instance, Robinson et al. (2012) used aversive stimuli such as monetary loss or sad faces to demonstrate that ATD enhanced punishment prediction and lowered resilience, whereas a higher level of TRP correlated with a lower punishment prediction and potentially a higher level of resilience to affective disorders. Therefore, the role of 5-HT in resilience may explain the results found in this study whereby ATD reduced pain tolerance, possibly via lowered resilience. However, to the best of our knowledge, there has not been any research outlining the role of 5-HT in the resilience of pain stimuli rather than monetary loss or sad faces prior to this study.

The decreased levels of TRP, and hence 5-HT, resulting in the sensitisation of the thermode-induced pain suggest that the serotonergic descending spinal-raphe projections have been altered. The action of 5-HT within this network can both inhibit and facilitate pain, and the outcome depends strongly on the type of 5-HT receptor subtype that is activated, such that anti-nociception is associated with activation of 5-HT_1A_, 5-HT_1B_, 5-HT_1D_ and 5-HT_7_ subtypes, whilst activation of 5-HT_2A_ and 5-HT_3_ is associated with pro-nociception (Ossipov et al. 2014). This study has shown that a global reduction in 5-HT results in sensitisation of pain and thus suggests that the activation of the anti-nociceptive receptor subtypes is reduced more than the pro-nociceptive receptor subtypes. The distribution of the anti-nociceptive receptor subtypes is widespread with high concentrations of 5-HT_1A/1B_ in the raphe nucleus and basal ganglia, whilst the pro-nociceptive 5-HT receptor subtypes are more prominent in the neocortex and not in the raphe nucleus (Burnet et al. 1995). Therefore, as the paradigm does not necessarily depend strongly on cognition via the higher cortical regions, it would be possible that the spinal-raphe projections and basal ganglia processing are more important in the processing of the threshold and tolerance responses to the thermode, and could explain why ATD resulted in sensitisation of pain response to the thermode. This could also explain why there was no significant effect seen in the CO_2_ laser attention paradigm.

The correlation analysis of TRP levels highlighted the quantitative relationship between the level of TRP depletion and changes in pain perception. This further strengthens that the concentration of 5-HT is related to the perception of pain. Furthermore, the fact that an acute decrease in 5-HT elicits a decrease of pain perception suggests that a persistent decrease of 5-HT over time could elicit a larger change in pain perception, which could be clinically presented as chronic pain. The long-term depletion of 5-HT may also play a part in the maladaptive neuroplastic changes that occur in the brain in chronic pain conditions. Low levels of 5-HT have been correlated with chronic pain disorders such as FM (Alnigenis and Barland 2001); however, results are variable possibly due to cerebrospinal fluid (CSF) providing a relatively indirect indication of changes in brain chemistry that is very dependent on CSF dynamics. In addition, it is widely known that a persistent reduction of 5-HT is linked with depression and anxiety disorders (Shopsin and Frank Feiner 1984), which have also been linked to the exacerbation and persistence of chronic pain (Jones and Brown 2017).

### Paradigm B: attention and distraction

Paradigm B investigated the use of laser stimuli and how the participant’s attentional state impacts pain perception. The investigation supported prior knowledge that distraction reduces the intensity of pain perceived (Boyle et al. 2008; Bantick et al. 2002; de Tommaso et al. 2008; Miron et al. 1989; Petrovic et al. 2000; Rémy et al. 2003; Tracey et al. 2002); however, this effect was only reported to be significant for high pain. Paradigm B was minimally affected by ATD such that there were no ATD-induced change in psychophysics or the distraction paradigm pain ratings. However, although not significant and only a trend was reported, it is interesting to note that ATD appeared to slightly increase the rating of high pain across both tasks. This would support the findings seen in paradigm A that 5-HT depletion results in sensitisation of pain. Nevertheless, paradigm B indicated that the distraction-induced analgesia was not altered by the acute depletion of 5-HT. Further investigation would be interesting to explore whether chronic depletion in TRP could elicit a stronger effect in the sensitisation of laser-evoked pain stimuli.

Nonetheless, previous research supports the notion that serotonin is involved in pain distraction. For instance, distraction analgesia coincides with increased activity within the posterior thalamus and PAG structures, as well as altered activity in pain matrix regions such as the insula cortex, the somatosensory cortices and the anterior cingulate cortex (ACC) (Bantick et al. 2002; Dunckley et al. 2007; Frankenstein et al. 2001). These aforementioned regions are all, directly or indirectly, potentially influenced by serotonergic activity (Ford et al. 2008; Lechin et al. 2005; Oke et al. 1997). Hence, it would be predicted that ATD could impact the typical function within these sites and result in an impaired distraction-induced analgesia. The reason that we have not seen any significant alteration in the distraction paradigm could be due to the short duration of the depletion or experimental design. For instance, the mathematical task was presented to the participant in both conditions to maintain a consistent visual fixation; however, this may have resulted in participants completing the mathematical questions unintentionally and diminishing the contrast between the distraction task results. Therefore, a modification of the current paradigm or an alteration in the type of distraction, such as auditory or a more complex task, could elicit a different outcome.

### Paradigm B: cognitive performance

Serotonergic neurotransmission is associated with general cognitive ability (Schmitt et al. 2006). We found that ATD impacted on cognitive and executive function during sensory discrimination and mathematical tasks. Following ATD, participants were poorer at discriminating between low- and high-pain stimulation. This highlights that cognitive processing may have been altered via the ATD. The significant decrease in the percentage correct calculations also highlights an impaired or altered level of cognition due to the ATD. These findings could be explained by serotonergic modulation of insula and somatosensory cortices which both receive serotonergic input as discussed in the preceding section.

### Methodological considerations

It is important to highlight that the small sample size of this study is a methodological limitation and further studies are required to confirm the reported findings.

### Relevance to current methods of treating pain disorders

There are numerous studies highlighting the use of SSRIs to treat chronic pain; however, the specific mechanism of its action is not completely understood and variation in effectiveness is well documented (Mika et al. 2013; McQuay et al. 1996). This study shows that 5-HT in humans has a potentially direct role in pain processing as we have successfully dissociated the effects from mood changes. The significant increase in sensitivity of the thermode temperature in this study implies that an increased level of 5-HT via the use of SSRIs would reverse the sensitivity seen due to low 5-HT. Additionally, the correlation analysis has indicated that the concentration of TRP, and thus 5-HT, is positively correlated to pain perception. The lack of difference seen in paradigm B, which involved both a different pain stimuli and cognitive demand, may help to explain the variability seen in the effectiveness of SSRIs used for chronic pain. The role of serotonin in pain is yet to be clearly defined and thus is likely to have multiple roles in pain processing. The findings in the current study could be explained on the basis of modulation of both the non-cognitive diffuse noxious inhibitory controls (DNIC) and cognitive components of nociceptive processing. Both of these could increase the resilience to pain in different ways. Recent studies have demonstrated a compensatory upregulation of opiate receptor binding in the brain in response to chronic pain that also correlates with increased pain resilience (increased pain tolerance) (Brown et al. 2008). The paper of Abbott and Young (1988) finding TRP depletion blocking opiate analgesia in humans suggests that both systems may be important modulators of pain resilience. The size of the effects on pain threshold and tolerance is, however, quite modest which is in keeping with the quite modest effect size seen in clinical pain studies of SSRIs. Therefore, the effectiveness of the SSRIs may depend on the type of chronic pain and individual cognitive and non-cognitive differences between patients. We should therefore probably not raise our expectations of the therapeutic effects of manipulation of this system on its own too much, but learn how individuals can best benefit from this without suffering untoward side effects. One possibility for the future, in relation to Seltzer et al. (1982), is potentially to prevent chronic pain by dietary supplementation by increasing the resilience of those at risk of chronic pain. This study has clearly shown that 5-HT has a role in modulating pain perception that is independent of mood state, and therefore, this study supports the beneficial use of SSRIs for treatment of chronic pain.

However, there is much debate regarding the involvement of 5-HTs in pain perception, and it is clearly not a one-directional relationship. 5-HT has been linked to several chronic pain conditions. However, the specific role of serotonin in chronic pain is conflicting such that low serotonin levels have been associated with fibromyalgia (Wolfe et al. 1997) whilst high serotonin has been linked to CRPS (Wesseldijk et al. 2008). In addition, there is evidence that neurodegeneration of serotonergic regions in Parkinson’s disease patients results in the development of chronic pain due to compromised descending pain pathways (Millan 2002; Hornung 2003; Braak et al. 2003; Halliday et al. 1990). The difference could be explained by the variance in the action of the 5-HT receptor subtypes, and a study has highlighted that both agonists and antagonists of specific receptor 5-HT subtypes are promising therapeutic targets (Bardin 2011).

Furthermore, a meta-analysis (McQuay and Moore 1997; McQuay et al. 1996) suggests that less specific tricyclic antidepressants (TCAs) and dual (5-HT and norepinephrine) reuptake inhibitors serotonin-norepinephrine reuptake inhibitors (SNRIs) are more effective in the treatment of pain. However, due to the negative side effects associated with TCAs, the most effective and safe serotonin-modulating analgesic appears to be SNRIs (Stahl et al. 2005). The effectiveness of TCAs and SNRIs suggests that the combined increase in both 5-HT and noradrenaline (NA) may be the most effective form of analgesia. As we know very little about the effects of NA on human nociceptive processing, this may be a relevant focus for future studies.

### Conclusion

In conclusion, this study has indicated a connection between the reduction of global 5-HT via ATD and an alteration of pain perception whilst dissociating these findings from altered mood state. The ATD significantly reduced both threshold and tolerance of the thermode temperature, demonstrating a clear role of 5-HT in pain perception. ATD did not significantly affect distraction-induced analgesia but did affect cognitive appraisal of the sensory-discriminatory aspects of pain. The study supports the understanding that low levels of 5-HT correlate to pain sensitisation and help to understand possible mechanisms of chronic pain states. It also helps to explain the variable effectiveness seen in the use of SSRIs for chronic pain. Future studies on 5-HT’s role in the cognitive and non-cognitive aspects of pain processing and chronic pain will be important to improve individual treatment choice for patients with chronic pain.
